# Multiobject Tracking of Wildlife in Videos Using Few-Shot Learning

**DOI:** 10.3390/ani12091223

**Published:** 2022-05-09

**Authors:** Jiangfan Feng, Xinxin Xiao

**Affiliations:** School of Computer Science and Technology, Chongqing University of Posts and Telecommunications, Chongqing 400065, China; s200231187@stu.cqupt.edu.cn

**Keywords:** camera trap, few-shot learning, wildlife management, animal behavior

## Abstract

**Simple Summary:**

Video recordings enable scientists to estimate species’ presence, richness, abundance, demography, and activity. The increasing popularity of camera traps has led to a growing interest in developing approaches to more efficiently process images. Advanced artificial intelligence systems can automatically find and identify the species captured in the wild, but they are hampered by dependence on large samples. However, many species rarely occur, such as endangered species, and only a few shot samples are available. Building on recent advances in deep learning and few-shot learning technologies, we developed a multiobject-tracking approach based on a tracking-by-detection paradigm for wildlife to improve multiobject-tracking performance. We hope that it will be beneficial to ecology and wildlife biology by speeding up the process of multiobject tracking in the wild.

**Abstract:**

Camera trapping and video recording are now ubiquitous in the study of animal ecology. These technologies hold great potential for wildlife tracking, but are limited by current learning approaches, and are hampered by dependence on large samples. Most species of wildlife are rarely captured by camera traps, and thus only a few shot samples are available for processing and subsequent identification. These drawbacks can be overcome in multiobject tracking by combining wildlife detection and tracking with few-shot learning. This work proposes a multiobject-tracking approach based on a tracking-by-detection paradigm for wildlife to improve detection and tracking performance. We used few-shot object detection to localize objects using a camera trap and direct video recordings that could augment the synthetically generated parts of separate images with spatial constraints. In addition, we introduced a trajectory reconstruction module for better association. It could alleviate a few-shot object detector’s missed and false detections; in addition, it could optimize the target identification between consecutive frames. Our approach produced a fully automated pipeline for detecting and tracking wildlife from video records. The experimental results aligned with theoretical anticipation according to various evaluation metrics, and revealed the future potential of camera traps to address wildlife detection and tracking in behavior and conservation.

## 1. Introduction

Biodiversity is an essential component and a key element in maintaining the stability of ecosystems. In the face of the current sharp decline in global biodiversity, it is urgent to take adequate measures to prevent and protect it. Wildlife monitoring and conservation that determine biodiversity patterns is a cornerstone of ecology, biogeography, and conservation biology. Therefore, monitoring animal habits and activity patterns during the rewilding training process is essential. Driven by advances in cheap sensors and computer-vision technologies for detecting and tracking wildlife, biodiversity research is rapidly transforming into a data-rich discipline. Video data have become indispensable in the retrospective analysis and monitoring of endangered animal species’ presence and behaviors. However, large-scale research is prohibited by the time and resources needed to process large data manually.

Recent technological advances in computer vision have led to wildlife scientists realizing the potential of automated computational methods to monitor wildlife. This ongoing revolution is facilitated by cost-effective mechanical high-throughput wildlife-tracking methods that generate massive high-resolution images across scales relevant to the ecological context in which animals perceive, interact with and respond to their environment. While applying existing tools is tempting, many potential pitfalls must be considered to ensure the responsible use of these approaches. For example, a large amount of data is required to train these deep-learning models accurately. However, because many species rarely occur, only a few shot samples are available; thus, the performance is typically low.

**Figure 1:**
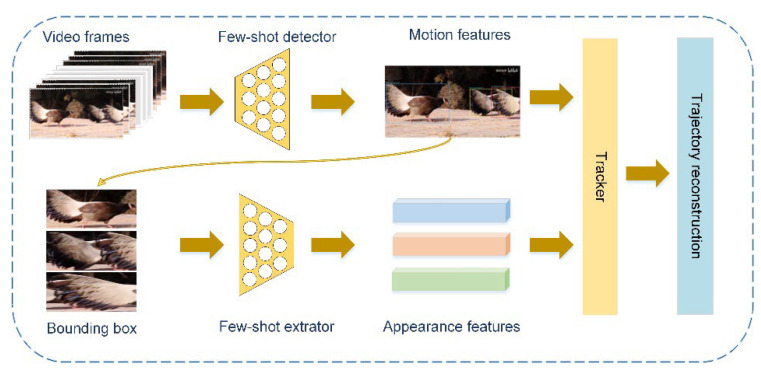
We aimed to obtain a few-shot multiobject-tracking model based on few-shot learning. In this framework, we used a few-shot object detector as the detector and a classification network trained based on the few-shot method as the feature extractor. In addition, we also designed a trajectory-reconstruction module to optimize the tracking result.

Few-shot learning aims to develop the ability to learn and generalize autonomously from a small number of samples. It can rapidly generalize to new tasks containing only a few samples with supervised information. Multiple recent publications have discussed this approach [1,2,3,4,5]. Generally, the research on multiobject tracking mainly focuses on how to improve the real-time performance of multiobject monitoring [6,7], how to better model the appearance information of the target [8,9,10,11], and how to associate targets efficiently [12,13,14,15]. Multiobject-tracking methods always follow the tracking-by-detection paradigm. In [7], this method was called separate detection and embedding (SDE). This means that the MOT system was broken down into two steps: (1) locating the target in single video frames; and (2) associating detected targets with existing trajectories. Another multi-object tracking learning paradigm, JDE, was also proposed. JDE jointly learned the detector and embedding model in a single deep network. In other words, the JDE method used a single network to output both the detection result and the corresponding appearance embeddings of the detected boxes. The SDE method used two separate networks to accomplish the above two tasks. JDE was closer to real-time performance, but the tracking accuracy was slightly worse than SDE. The small-sample object-detector performance was not as good as that of YOLO [16,17,18,19], Faster R-CNN [20], and other general object detectors [21,22]. In the object detection of each frame, there will be missed detection, which significantly affects the effect of the multiobject-tracking task. Therefore, to ensure the performance effect of a multiobject-tracking model driven by a small amount of data, in addition to selecting the SDE paradigm, we also proposed a trajectory reconstruction module in the data association part to further optimize the tracking accuracy, as shown in Figure 1.

The research hotspots of multiobject tracking under the tracking-by-detection paradigm always have the following two aspects: (1) a more accurate detection of targets in complex environments; and (2) the ability to deal with long-term occlusion and short-term occlusion problems and to associate targets more accurately. Some previous works [23,24,25] showed that a multiobject-tracking approach could achieve a state-of-the-art performance when used together with a robust object detector. They used Kalman filtering to predict and update trajectories [23] and proposed an extension [24]. In addition to considering the motion features above, the apparent features of the target were also considered. Feichtenhofer et al. introduced correlation features representing object cooccurrences across time to aid the ConvNet during tracking. Moreover, they linked the frame-level detections based on across-frame tracks to produce high-accuracy detections at the video level [25].

The primary purpose of data association is to match multiple targets between frames, including the appearance of new marks, the disappearance of old targets, and the identity matching of targets between consecutive frames. Many approaches formulated the data-association process as various optimization problems [12,13]. The former mapped the maximum a posteriori (MAP) data-association problem to cost-flow networks with nonoverlapping constraints on trajectories. A min-cost flow algorithm found the optimal data association in the network. The latter believed that re-identification only by appearance was not enough, and long-distance object reproduction was also worthy of attention. They proposed a graph-based formulation that linked and clustered person hypotheses over time by solving an instance of a minimum cost lifted multicut problem. Some works, such as [26,27], emphasized improving the features used in data association. They proposed dual matching attention networks with spatial and temporal attention mechanisms [26]. The spatial attention network generated dual spatial attention maps based on the cross-similarity between each location of an image pair, making the model more focused on matching common regions between images. The temporal attention module adaptively allocated different levels of attention to separate samples in the tracklet to suppress noisy observations. To obtain a higher precision, they also developed a new training method with ranking loss and regression loss [27]. The network considered the appearance and the corresponding temporal frames for data association.

Conceptually, tracking technologies using computer vision permit high-resolution snapshots of the movement of multiple animals and can track nontagged individuals, but they are less cost-effective, are usually limited to specific scenarios, and make individual identification challenging. In contrast, here we provide a fully automated computational approach to tracking tasks for wildlife by combining few-shot learning with multiobject tracking to detect, track, and recognize nature. It could represent a step-change in our use of extensive video data from the wild to speed up the procedure for ethologists to analyze biodiversity for research and conservation in the wildlife sciences. This approach represents an automated pipeline for recognizing and tracking species in the wild. Our main contributions can be summarized as follows:

- We combined few-shot learning with a multiobject-tracking task. To the best of our knowledge, the multiple automated object-tracking frameworks based on few-shot learning are being proposed for the first time.
- Our approach effectively merged the richness of deep neural network representations with few-shot learning that paves the way for robust detection and tracking of wildlife, which can be adaptive for unknown scenarios by data augmentation.
- A trajectory reconstruction module was proposed to compensate for the shortcomings of the few-shot object-detection algorithm in the multiobject-tracking tasks, especially in monitoring wildlife.

## 2. Materials and Methods

### 2.1. Architecture Overview

While camera traps have become essential for wildlife monitoring, they generate enormous amount of data. The fundamental goal of using intelligent frameworks in wildlife monitoring is automated analyses of behaviors, interactions, and dynamics, both individual and group. For example, sampling the quantity of species’ complex interactions for network analysis is a significant methodological challenge. Early approaches require capturing subjects and are labor-intensive. Their application may be location-specific, and the recorded data typically lacks contextual visual information. In this work, we instead sought to learn the unstrained dynamics and be sensitive to the presence of various locations and groups. The aim was to propose a cost-effective wildlife-tracking approach that generated massive high-resolution video records across scales relevant to the ecological context in which animals perceive, interact with and respond to their environment.

**Figure 2:**
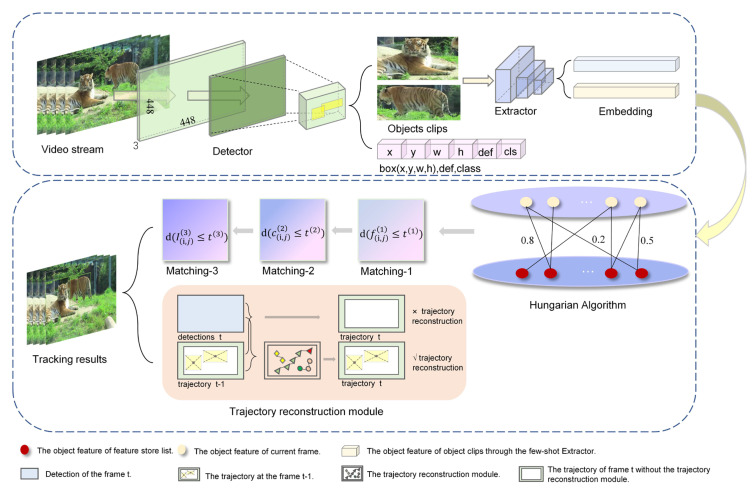
The architecture of our proposed few-shot tracker framework: Few-MOT. It consisted of a detection process and a tracking process. The detection process followed a few-shot object detector that directly regressed the objectness score (def), bounding box location (x,y,w,h), and classification score (cls). The tracking process included a few-shot feature-extraction network (Extractor), a matching module, and a trajectory-reconstruction module. The extractor was responsible for extracting the features of each object clip. The matching module then performed the association of targets between frames, and if they met the reconstruction criteria, they were constructed by the trajectory-reconstruction module. The details of this module will be explained in the methods section.

Figure 2 shows the overall design of the proposed MOT framework, called Few-MOT, which followed the tracking-by-detection paradigm, but without requiring large amounts of training data. An input video frame first underwent a forward pass through a few-shot object detector and a few-shot feature extractor to obtain motion and appearance information. Finally, we followed [24] and made improvements to solve the association problem for a few-shot setting. The upgrades included two parts: (1) a three-stage matching process including cascade matching, central-point matching, and IoU matching; and (2) a trajectory-reconstruction module to compensate for few-shot object detection.

### 2.2. Few-Shot Detection Module

Most object-detection approaches rely on extensive training samples. These requirements substantially limit their scalability to open-ended accommodation of novel classes with limited labeled training data. In general, the detection branch of multiobject tracking is the state-of-the-art of the object-detection field. Given the extreme scarcity of endangered animal scenes, we had very few samples available. This paper addresses these problems by offering a few-shot object detection with spatial constraints to localize objects in our multiobject-tracking framework. Few-shot object detection only requires a k-shot training sample, and its performance is better than that of the general detector under the same premise.

First, a note that in few-shot learning, we defined a large number of samples as the base, with their counterparts as the novel. In this paper, the novel class refers to the endangered animal class. Our proposed few-shot object-detection method allowed for few-shot learning in different scenarios with spatial dependencies while adapting to a dynamically changing environment during the detection process. It exploited a set of objects and environments that were processed, composed, and affected by each other simultaneously, instead of being recognized individually. Considering the geographical correlation between species and environmental factors, we thus proposed spatial constraints during the data augmentation. The images were first separated from the front and back views using the pretrained saliency network U2-Net [28]. Then, the pretrained image-inpainting network CR-Fill [29] repaired the missing parts. Finally, the foreground and background, which were separated, were blended and combined into a new sample. We used a perceptual hashing algorithm for spatial constraints during the combinations that did not correspond to the actual situation. For example, an event with a zero probability, such as a giant panda in the sky, would be misleading for training the object-detection model. After the above-constrained data expansion, the samples were learned from each other. The training of the few-shot object-detection task was performed based on a feature-reweighting method [30].

The perceptual hash algorithm pHash reduced the image frequency by the discrete cosine transform (DCT) and then matched similar images by calculating the Hamming distance. The algorithm proceeded as follows: (1) reduce the image to 32 ∗ 32; (2) convert the image to a grey-scale image; (3) calculate the DCT and DCT mean; (4) perform image pairing to calculate the Hamming distance. The equations to calculate the DCT and Hamming distance are shown in Equations (1)–(3) below:

(1) $$F=AfA^{T},$$

(2) $$A\left(i,j\right)=c\left(i\right)\cos\left[\frac{\left(j+0.5\right)\pi }{N}i\right],$$

**Figure 3:**
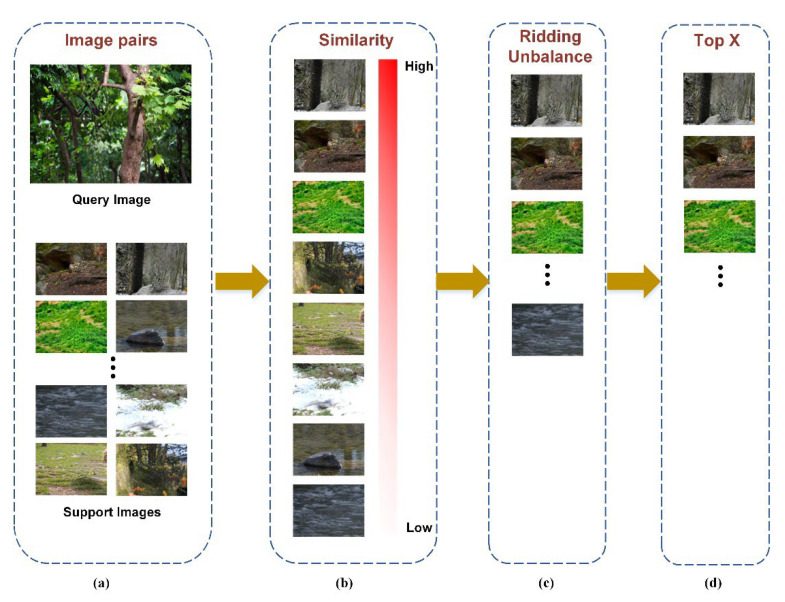
Filtering similar background processes: (**a**) calculating the Hamming distance between pairs of images; (**b**) sorting them in descending order by similarity; (**c**) removing the remarkably similar samples to ridding unbalance; (**d**) selecting the top 60% of reasonable samples, as those that could be subsequently blended for the front and back views.

(3) d(x,y)=∑x[i]⊕y[i],

This analysis can be extended toward a graphical representation (Figure 3).

### 2.3. Learning More Robust Appearance Embedding Based on Few-Shot Learning

There is an appearance metric-learning problem in a multiobject-tracking task, and the aim is to learn an embedding space where instances of the same identity are close while instances of different identities are far apart. The metric-learning problem is often defined as a re-identification task in multiobject tracking, mainly aimed at a single category; i.e., pedestrians or vehicles. For example, person re-identification aims at searching for persons across multiple nonoverlapping cameras. The task of Re-ID in this approach shares similar insights with the Re-ID for persons. When presented with an animal-of-interest (query) in video records, an animal Re-ID tells whether this animal has been observed in another place (time). In particular, we tracked nonsingle classes, and each class had very little training data. Thus, we trained the embedding learning process on the few-shot classification task.

Typically, few-shot classification approaches include optimization-based, model-based, and metric-based methods. Since our goal was not to classify but to train a feature learner based on the classification task and its feature map to the target, we performed descriptions of categories and changes in behavior. Thus, directly using a few-shot classification network for training was not applicable. We used elastic-distortion data augmentation to ensure the features had single information. Elastic distortion changed the posture of the target, allowing changes in behavior to be focused and adapted to our eventual tracking task. Because the target was moving and the pose of the same target was constantly changing in the video stream, this variation affected the recognition rate of the target identity during the tracking process.

**Figure 4:**
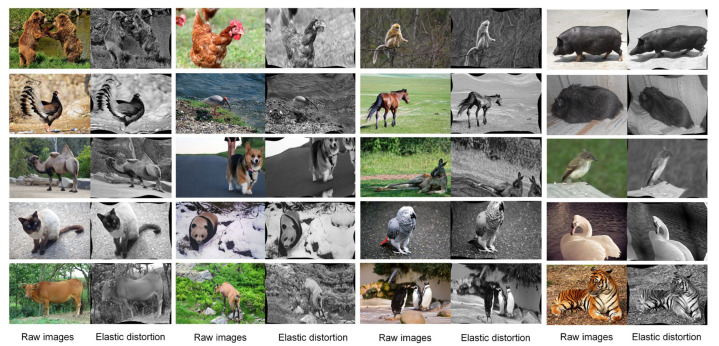
Example comparison of the EAOD dataset after elastic distortion. Each target was appropriately distorted without distorting the image. In this way, the diversity of target poses was enriched.

Firstly, the affine transformation of the image was performed to obtain a random displacement field generated by each pixel of the image. Then, we convolved the random displacement field with N(0,δ), which obeyed the Gaussian distribution, and multiplied the random displacement field by the control factor α, where δ controlled the smoothness of the image and α controlled the strength of the image deformation. We set δ to 0.07 and α to 5. The experimental results suggested that these parameter values enriched the target pose without distorting the image. Figure 4 shows a partial example of the processed image.

We imitated the approach used in [31] in our training process, using self-supervision and regularization techniques to learn generic representations suitable for few-shot tasks. Firstly, we used a pretext task called rotation to construct the self-supervised task on the base classes. In the self-supervised task, the input image was rotated by r degrees and $r\in C_{R}=\left\{0{}^{\circ},90{}^{\circ},180{}^{\circ},270{}^{\circ}\right\}$. The secondary purpose of the model was to predict the amount of rotation applied to the image. An auxiliary loss was added to the standard classification loss in the image classification setting to learn the generic representation. Secondly, fine-tuning with a manifold mixup was conducted on the base classes and endangered classes for a few more epochs. The manifold mixup provided a practical way to flatten a given class of data representations into a compact region. The loss function of the first stage is given by:

(4) $$L_{rot}=\frac{1}{\left|C_{R}\right|}*\underset{x\in D_{b}}{\sum }\underset{r\in C_{R}}{\sum }L\left(c_{W_{r}}\left(f_{\theta }\left(g{\left(x\right)}^{r}\right)\right),r\right),$$

(5) $$L_{class}=E_{\left(x,y\right)\in D_{b},r\in C_{R}}\left[L\left(g{\left(x\right)}^{r},y\right)\right]\;,$$

where $L_{rot}$ denotes the self-supervision loss, and $L_{class}$ denotes the classification loss. The loss function of the fine-tuning stage is given by:

(6) $$L_{mm}=E_{\left(x,y\right)\in D_{b}}\left[L\left(Mix_{\lambda }\left(f_{\theta }^{l}\left(x\right),f_{\theta }^{l}\left(x^{\prime }\right)\right),Mix_{\lambda }\left(y,y^{\prime }\right)\right)\right],$$

(7) $$Mix_{\lambda }\left(a,b\right)=\lambda *a+\left(1-\lambda \right)*b\;,$$

In addition, we used the input data x and $x^{\prime }$ with corresponding feature representations at layer l given by $f_{\theta }^{l}\left(x\right)$ and $f_{\theta }^{l}\left(x^{\prime }\right)$, respectively.

### 2.4. Association Module

Considering that the current association modules were all associated with the conventional multiobject-tracking task and were not applied to the multiobject-tracking task with a few-shot setting, it was inevitable that there were some shortcomings. To fit the Few-MOT module to the MOT-EA dataset, we made some improvements with the DeepSORT association algorithm.

#### 2.4.1. Three-Stage Matching

In addition to cascade matching and IoU matching, we added a central-point matching, which helped to alleviate the mismatched detection boxes and tracks due to an excessive intersection ratio. The IoU matrix $iou_{j,i}$ was calculated as the intersection-over-union (IoU) distance between every detection and object pair.

(8) $$iou_{j,i}=\frac{Area\left(track_{j}\right)\cap Area\left(dec_{i}\right)}{Area\left(track_{j}\right)\cup Area\left(dec_{i}\right)},\;$$

where $Area\left(track_{j}\right)$ is the area of $track_{j}$, and $Area\left(dec_{i}\right)$ represents the area of $dec_{i}$.

**Figure 5:**
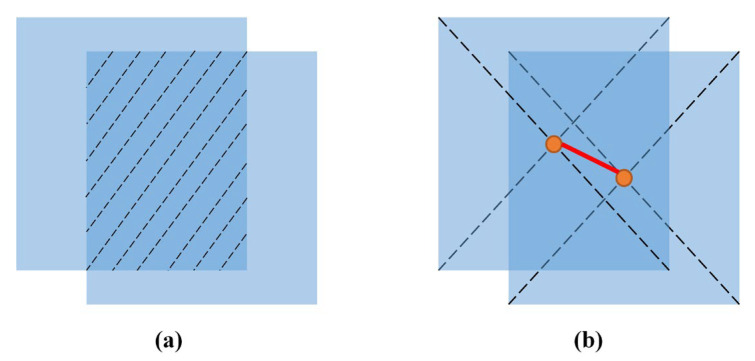
(**a**) IoU matching; (**b**) central point matching.

The central-point matrix $center_{j,i}$ was calculated as the central-point distance between every detection and track pair. Figure 5 illustrates the difference between center-point matching and IoU matching.

(9) $$center_{j,i}=dis\left(center\left(track_{j}\right),center\left(dec_{i}\right)\right),\;$$

where $center\left(track_{j}\right)$ and $center\left(dec_{i}\right)$ are the central-point of the track and detection, respectively.

During the experiment, we found that if we only used cascade matching and central-point matching in the matching stage, it did help to reduce ID switching, but at the same time, it was accompanied by an increase in missed targets. Thus, we worked together on IoU matching and central-point matching and designed the following trajectory-reconstruction module to alleviate this problem. In the MOT-EA dataset, we measured the above two matching strategies using the two indicators for FN and FP, and found that three-stage matching was the best matching strategy. A further discussion of the ablation experiment reveals more details.

#### 2.4.2. Trajectory-Reconstruction Module

We found an excessive amount of missed detection cases in the tracking process given in the previous section, which damaged the tracking effect. In addition, the performance of the few-shot detector was not as good as YOLO, Faster R-CNN, and other general object detectors. The target was then lost in the video stream. However, according to [32], the tracking accuracy of multiple objects can be written as:

(10) $$MOTA=1-\frac{FN+FP+IDSW}{GT}\in \left(-\infty ,1\right],\;$$

where FN is false negatives (the sum of missing amounts in the entire video), FP is false positives (the sum of the number of false positives in the entire video), IDSW is the ID switch (the total number of ID switches), and GT is the number of the ground truth objects. The object-detection accuracy significantly affected the tracking accuracy, so we designed a trajectory-reconstruction module to deal with the above problems. This module compensated for the lack of a few-shot detector.

**Figure 6:**
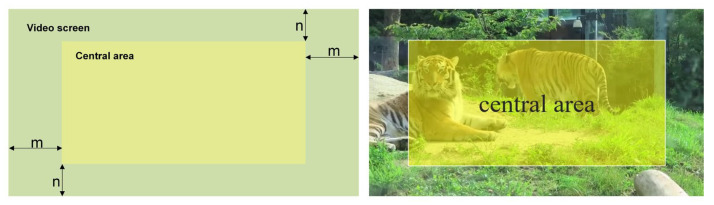
Schematic diagram of the division of the central area. The diagram on the left is an abstract representation, where we defined the central area as a fixed-scale area at the boundary of the video screen. The real situation is shown in the diagram on the right.

First, we specified the central region, as shown in Figure 6 below. Then, if there was no trajectory and the detection box was successfully matched in frame T, we judged the central-point position of the track in frame T-1. If the central point of the bounding box in frame T-1 was located in the central area, we reconstructed the track of frame T-1 to frame T under the present conditions. We allowed the reconstruction of five consecutive frames because the object’s position usually changed slightly in five consecutive frames. The box of frame T-1 could still locate the object’s position in the subsequent four frames.

## 3. Results

### 3.1. Implementation Details

This framework was written in Python with PyTorch support. First, when training the feature extractor of Few-MOT, we converted the EAOD private object-detection dataset into an image-classification dataset for training. WRN-28-10 [33] was used as the backbone, and the elastic-distortion data-augmentation strategy enhanced the feature robustness of animals in various poses. Then, in the design of the trajectory-reconstruction module, we found through several experiments that when the allowable reconstruction threshold was set to less than 5, there were too many missed trajectories. When the setting was greater than 5, there were too many false trajectories, which reduced the tracking effect. Therefore, we set the threshold for the maximum number of frames allowed to be continuously reconstructed to 5.

### 3.2. Datasets and Evaluation Metrics

1. Datasets: Currently, there is no multiobject-tracking dataset for endangered animals, so we created the MOT-EA multiobject-tracking dataset in the format of MOT-16 [34]. The dataset included five endangered species: brown-eared pheasant, crested ibis, giant panda, golden snub-nosed monkey, and tiger. Each video was 10 to 20 s in length. Details are shown in Table 1 below.
2. Evaluation Metrics: Following the benchmarks, we evaluated our work using [32]. MOTA and IDF1 are considered the two most important among all metrics. MOTA is an indicator to measure the accuracy of multiobject tracking. Mostly, it considers the matching errors of objects in the tracking process. According to FP, FN, and IDs, MOTA gives a very intuitive measure of the tracker performance, which is independent of the accuracy of object detection. The IDF1 considers the ID accuracy rate and the ID recall rate comprehensively, and considers the ID information more than MOTA. However, IDF1 cannot reflect the phenomenon of ID switch. This is shown in Equations (10) and (11) below. A robust tracking system should show good scores for both MOTA and IDF1.
  (11) $$IDF1=\frac{2IDTP}{2IDTP+IDFP+IDFN\;},\;$$

### 3.3. Experimental Results

Here, we evaluated our system using the MOT-EA dataset. Table 2 shows the tracking performance of our framework on the five endangered categories. Furthermore, we compared the same few-shot object detector with multiple trackers, as shown in the first four rows of Table 3. On the other hand, the general detector YOLOv4 was used for comparison, as shown in row 5 of Table 3. The specific performance of the five methods in Table 3 on the MOT-EA dataset is supplemented in Appendix A Table A1, Table A2, Table A3, Table A4 and Table A5. The results showed that our framework outperformed many previous approaches with small data samples. Both the MOTA and IDF1 scores were in the leading position for MOT-EA. We believe that the following results were obtained because the general detector could not achieve a good detection effect with a small amount of data, which significantly affected the tracking. In addition, the tracker we designed was more suitable for this scenario. It is more robust to various morphological changes in animals, and more targeted to insufficient learning caused by a small amount of data.

**Figure 7:**
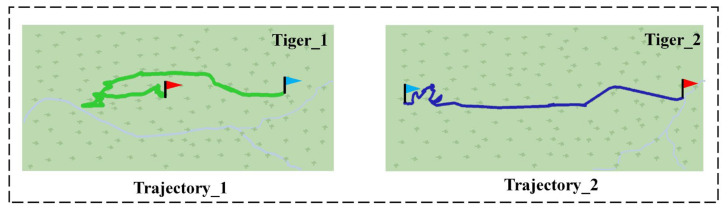
Tracks 1 and 2 are the respective tracks recorded for two tigers, with the red flag representing the starting point and the blue flag representing the endpoint.

**Figure 8:**
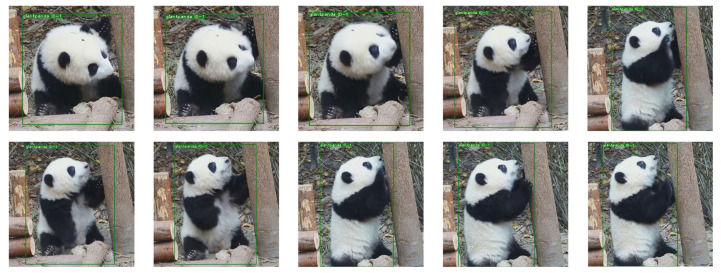
Tracking example of a giant panda.

**Figure 9:**
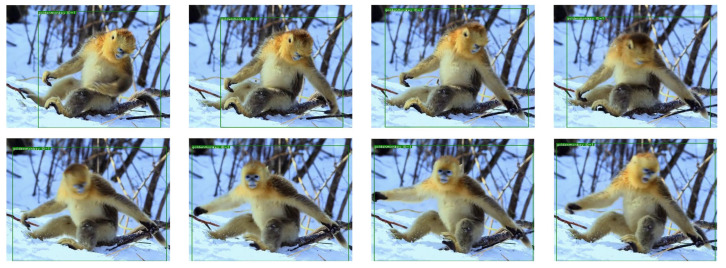
Tracking example of a golden snub-nosed monkey.

Two example trajectories of two tigers using the Few-MOT model are shown in Figure 7 below. Our model made it possible to track the targets and plot the movements. We could record the basic trajectories of the endangered animals within the monitoring area. Furthermore, we could also use the trajectories to analyze the areas where the targets were active, determine whether they were involved and the interaction between different targets, etc. In addition, the tracking processes of a giant panda and a golden snub-nosed monkey are shown in Figure 8 and Figure 9, respectively. The targets were continuously located during this process and maintained unique identity IDs.

### 3.4. Ablation Study and Discussion

Here, we discuss the impact of the three parts of the three-stage matching and elastic-distortion data-augmentation strategy and the trajectory-reconstruction module. First, we performed ablation experiments on the MOT-EA dataset for the matching module. The two stages included cascade matching and central matching. The three stages included cascade matching, central matching, and IoU matching. As shown in Table 4, the three-stage matching showed improvement in the cases of false and missed detections.

Table 5 shows the impacts of the two parts of the elastic-distortion data-augment strategy and the trajectory-reconstruction module. The baseline model (row 1 in Table 5) consisted of a few-shot detector and an unmodified tracker. The other experimental results in Table 5 shared the same set of few-shot detectors, except for the feature learner’s training process and the tracker’s association module. The results indicated that the feature stability brought by the elastic-distortion data-enhancement strategy slightly improved the MOTA index. However, the more significant effect stemmed from the proposal of the trajectory-reconstruction module. This module handled both false and missed targets well in the tracking process. According to Equation (10), it led to a significant improvement in the MOTA.

**Figure 10:**
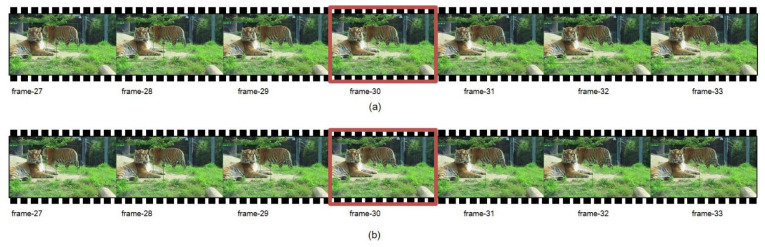
Tracking sequence before and after frame 30: (**a**) performance without trajectory-reconstruction module; (**b**) performance with the trajectory-reconstruction module.

Figure 10 shows a small segment of the performance of the trajectory reconstruction module during the tracking process. In comparison, we can find that the target lost in the 30th frame was reconstructed. This module made the trajectory of the target more complete.

## 4. Discussion

So-called “big data” approaches are not limited to technical fields because the combination of large-scale data collection and processing techniques can be applied to various scientific questions. Meanwhile, it has never been more critical to keep track of biodiversity than over the past decade, as losses and declines have accelerated with ongoing development. However, multiobject tracking is complicated, with experts relying on human interactions and specialized equipment. While cheap camera sensors have become essential for capturing wildlife and their movements, they generate enormous amounts of data, and have become a prominent research tool for studying nature. Machine- and deep-learning methods hold promise as efficient tools to scale local studies to a global understanding of the animal world [38]. However, the detection and tracking of the target animals are challenging, essentially because the data obtained from wild species are too sparse.

Our deep-learning approach detected and tracked the target animals and produced spatiotemporal tracks that following multiple objects through few-shot learning to alleviate instance imbalance and insufficient sample challenges. This study demonstrated how incorporating track methods, deep learning, and few-shot learning can be a research tool for studying wild animals. Turning now to its limitations, we note that our approach heavily relied on the prominent parts’ detection performance, and easily failed to track infant animals.

## 5. Conclusions

In this work, we introduced Few-MOT for wildlife to embed uncertainty into designing a multiobject-tracking model by combining the richness of deep neural networks with few-shot learning, leading to correctable and robust models. The approach systematically provided a fully automated pipeline framework to integrate the few-shot learning method with deep neural networks. Instead of a discriminative model, a spatial-constraints model was created. Furthermore, a trajectory-reconstruction module was also proposed to compensate for the shortcomings of the few-shot object detection. Our model demonstrated the efficacy of using few-shot architectures for biological application: the automated recognition and tracking of wildlife. Unlike older, data-rich automation methods, our method was entirely based on deep learning with few shots. It also improved previous deep-learning methods by combining few-shot learning with a multiobject-tracking task. It also provided a rich set of examples by incorporating contextual details of the environment, which can be valuable for few-shot learning efficiency, especially in wildlife detection and tracking.

The data explosion that has come with the widespread use of camera traps poses challenges while simultaneously providing opportunities for wildlife monitoring and conservation [39]. Tracking animals is essential in animal-welfare research, especially when combined with physical and physiological parameters [40,41,42]. It is also challenging to curate datasets large enough to train tracking models. We proposed a deep-learning framework named Few-MOT to track endangered animals based on a few-shot-learning and tracking-by-detection paradigm. It could record the daily movements of the target being tracked, marking areas of frequent activity and other information that could be used for further analysis. This framework offered a few-shot object detection with spatial constraints to localize objects and a trajectory-reconstruction module for a better association. The experimental results showed that our method performed better on the few-shot multiobject-tracking task. Our new datasets open up many opportunities for further research on multiobject tracking. There were some limitations to our study, notably that the detector could detect a nonexistent target in the wrong place when the surroundings were extremely similar to the target. Future work should investigate how multiple variables, such as the features of the training dataset and different network architectures, affect performance. Furthermore, a key driver in the advancement of intelligent video systems for wildlife conservation will be the increasing availability of datasets for sufficient species, and open-source datasets should also be proposed in the future.

## Figures and Tables

**Table 1:** Detail of MOT-EA dataset.

Class	Duration (s)
Brown-eared pheasant	13:26
Crested ibis	16:24
Giant panda	20:00
Golden snub-nosed monkey	10:21
Tiger	14:29

**Table 2:** Results of the proposed MOT framework for MOT-EA.

Class	IDF1	IDP	IDR	FP ↓	FN ↓	IDs ↓	MOTA	MOTP
Tiger	59.30%	71.7%	50.5%	66	281	2	52.10%	0.287
Golden snub-nosed monkey	95.50%	99.4%	91.9%	2	28	0	91.40%	0.224
Giant panda	72.10%	83.8%	63.3%	96	295	2	51.50%	0.285
Crested ibis	62.40%	74.1%	53.8%	0	253	7	71.90%	0.278
Brown-eared pheasant	34.10%	50.7%	25.7%	46	634	12	42.00%	0.273
OVERALL	64.68%	75.94%	57.04%	210	1491	23	61.78%	0.27

↓ means the smaller the better.

**Table 3:** Comparison with the same few-shot detector and YOLOv4.

Class	IDF1	IDP	IDR	FP ↓	FN ↓	IDs ↓	MOTA	MOTP
BYTETrack [35]	59.50%	76.38%	49.04%	187	1739	14	53.86%	0.22
SORT [23]	29.26%	45.76%	21.66%	92	2201	85	41.64%	0.211
IoU-tracker [36]	15.70%	23.82%	12.12%	143	2330	210	37.40%	0.215
V-IoU-tracker [37]	38.56%	62.14%	29.24%	80	2192	27	48.34%	0.212
YOLOv4 [19] + DeepSORT [24]	35.80%	57.06%	27.62%	76	2436	76	40.46%	0.227
Ours	64.68%	75.94%	57.04%	210	1491	23	61.78%	0.27

↓ means the smaller the better.

**Table 4:** Performance comparison for the matching module with different methods.

Method	FP ↓	FN ↓
Two stages	337	1627
Three stages	210	1491

↓ means the smaller the better.

**Table 5:** Effects of using the elastic-distortion data-augmentation strategy and trajectory-reconstruction module for tracking.

Augment	Trajectory Reconstruction	IDs ↓	MOTA
-	-	30	52.58%
√	-	33	52.72%
√	√	23	61.78%

↓ means the smaller the better.

## Data Availability

The data used to support the findings of this study are available from the corresponding author upon request.

## Appendix A

**Table A1:** Results of the same few-shot object detector in our model combined with BYTETrack tracker on the MOT-EA dataset.

Class	IDF1	IDP	IDR	FP ↓	FN ↓	IDs ↓	MOTA	MOTP
Tiger	59.20%	80.8%	46.7%	52	359	1	43.40%	0.189
Golden snub-nosed monkey	83.70%	97.7%	73.2%	2	89	1	73.50%	0.173
Giant panda	28.70%	36.4%	23.7%	102	385	7	39.10%	0.23
Crested ibis	77.70%	96.9%	64.9%	6	312	1	65.50%	0.255
Brown-eared pheasant	48.20%	70.1%	36.7%	25	594	4	47.80%	0.253
OVERALL	59.50%	76.38%	49.04%	187	1739	14	53.86%	0.22

↓ means the smaller the better.

**Table A2:** Results of the same few-shot object detector in our model combined with SORT tracker on the MOT-EA dataset.

Class	IDF1	IDP	IDR	FP ↓	FN ↓	IDs ↓	MOTA	MOTP
Tiger	28.20%	50.2%	19.6%	25	468	14	30.40%	0.183
Golden snub-nosed monkey	28.00%	37.9%	22.2%	2	146	14	53.30%	0.174
Giant panda	16.70%	24.9%	12.6%	55	456	21	34.40%	0.214
Crested ibis	30.10%	43.8%	22.9%	1	442	20	49.90%	0.247
Brown-eared pheasant	43.30%	72.0%	31.0%	9	689	16	40.20%	0.237
OVERALL	29.26%	45.76%	21.66%	92	2201	85	41.64%	0.211

↓ means the smaller the better.

**Table A3:** Results of the same few-shot object detector in our model combined with IoU-tracker tracker on the MOT-EA dataset.

Class	IDF1	IDP	IDR	FP ↓	FN ↓	IDs ↓	MOTA	MOTP
Tiger	14.90%	25.4%	10.6%	41	466	47	23.90%	0.197
Golden snub-nosed monkey	27.30%	32.3%	23.6%	1	94	20	66.90%	0.169
Giant panda	7.70%	10.6%	6.0%	75	424	37	33.90%	0.219
Crested ibis	19.30%	31.0%	14.1%	2	507	50	39.60%	0.251
Brown-eared pheasant	9.50%	19.8%	6.3%	24	839	56	23.00%	0.237
OVERALL	15.70%	23.82%	12.12%	143	2330	210	37.40%	0.215

↓ means the smaller the better.

**Table A4:** Results of the same few-shot object detector in our model combined with V-IoU-tracker tracker on the MOT-EA dataset.

Class	IDF1	IDP	IDR	FP ↓	FN ↓	IDs ↓	MOTA	MOTP
Tiger	44.30%	78.9%	30.8%	0	444	2	38.70%	0.191
Golden snub-nosed monkey	45.80%	48.4%	43.5%	0	35	3	89.00%	0.172
Giant panda	12.10%	15.9%	9.7%	77	391	14	40.60%	0.22
Crested ibis	45.90%	89.9%	30.8%	1	609	1	33.90%	0.24
Brown-eared pheasant	44.70%	77.6%	31.4%	2	713	7	39.50%	0.238
OVERALL	38.56%	62.14%	29.24%	80	2192	27	48.34%	0.212

↓ means the smaller the better.

**Table A5:** Results of the YOLOv4 detector combined with DeepSORT tracker on the MOT-EA dataset.

Class	IDF1	IDP	IDR	FP ↓	FN ↓	IDs ↓	MOTA	MOTP
Tiger	18.60%	43.4%	11.8%	6	536	17	23.20%	0.233
Golden snub-nosed monkey	69.40%	80.8%	60.8%	0	86	7	73.20%	0.202
Giant panda	30.90%	41.0%	24.8%	62	383	24	42.20%	0.222
Crested ibis	35.40%	60.3%	25.1%	3	543	5	40.40%	0.213
Brown-eared pheasant	24.70%	59.8%	15.6%	5	888	23	23.30%	0.268
OVERALL	35.80%	57.06%	27.62%	76	2436	76	40.46%	0.227

↓ means the smaller the better.

## References

[1] Vinyals O., Blundell C., Lillicrap T., Kavukcuoglu K., Wierstra D. Matching networks for one shot learning. Proceedings of the Advances in Neural Information Processing Systems 29 (NIPS 2016).

[2] Snell J., Swersky K., Zemel R. Prototypical networks for few-shot learning. Proceedings of the Advances in Neural Information Processing Systems 30 (NIPS 2017).

[3] Wang Y., Yao Q. (2019). Few-shot learning: A survey. arXiv.

[4] Chen W.-Y., Liu Y.-C., Kira Z., Wang Y.-C.F., Huang J.-B. (2019). A closer look at few-shot classification. arXiv.

[5] Oreshkin B., Rodríguez López P., Lacoste A. (2018). Tadam: Task dependent adaptive metric for improved few-shot learning. Adv. Neural Inf. Processing Syst..

[6] Du Y., Yan Y., Chen S., Hua Y.J.N. (2020). Object-adaptive LSTM network for real-time visual tracking with adversarial data augmentation. Neurocomputing.

[7] Wang Z., Zheng L., Liu Y., Li Y., Wang S. Towards real-time multi-object tracking. Proceedings of the European Conference on Computer Vision.

[8] Fan H., Ling H. Siamese cascaded region proposal networks for real-time visual tracking. Proceedings of the IEEE/CVF Conference on Computer Vision and Pattern Recognition.

[9] Kim C., Fuxin L., Alotaibi M., Rehg J.M. Discriminative appearance modeling with multi-track pooling for real-time multi-object tracking. Proceedings of the IEEE/CVF Conference on Computer Vision and Pattern Recognition.

[10] Wang Q., Zheng Y., Pan P., Xu Y. Multiple object tracking with correlation learning. Proceedings of the IEEE/CVF Conference on Computer Vision and Pattern Recognition.

[11] Pang J., Qiu L., Li X., Chen H., Li Q., Darrell T., Yu F. Quasi-dense similarity learning for multiple object tracking. Proceedings of the IEEE/CVF Conference on Computer Vision and Pattern Recognition.

[12] Zhang L., Li Y., Nevatia R. Global data association for multi-object tracking using network flows. Proceedings of the 2008 IEEE Conference on Computer Vision and Pattern Recognition.

[13] Tang S., Andriluka M., Andres B., Schiele B. Multiple people tracking by lifted multicut and person re-identification. Proceedings of the IEEE Conference on Computer Vision and Pattern Recognition.

[14] Dai P., Weng R., Choi W., Zhang C., He Z., Ding W. Learning a proposal classifier for multiple object tracking. Proceedings of the IEEE/CVF Conference on Computer Vision and Pattern Recognition.

[15] Saleh F., Aliakbarian S., Rezatofighi H., Salzmann M., Gould S. Probabilistic Tracklet Scoring and Inpainting for Multiple Object Tracking. Proceedings of the IEEE/CVF Conference on Computer Vision and Pattern Recognition.

[16] Redmon J., Divvala S., Girshick R., Farhadi A. You only look once: Unified, real-time object detection. Proceedings of the IEEE Conference on Computer Vision and Pattern Recognition.

[17] Redmon J., Farhadi A. YOLO9000: Better, faster, stronger. Proceedings of the IEEE Conference on Computer Vision and Pattern Recognition.

[18] Redmon J., Farhadi A. (2018). Yolov3: An incremental improvement. arXiv.

[19] Bochkovskiy A., Wang C.-Y., Liao H.-Y.M. (2020). Yolov4: Optimal speed and accuracy of object detection. arXiv.

[20] Ren S., He K., Girshick R., Sun J. (2016). Faster r-cnn: Towards real-time object detection with region proposal networks. Adv. Neural Inf. Processing Syst..

[21] Lin T.-Y., Goyal P., Girshick R., He K., Dollár P. Focal Loss for Dense Object Detection. Proceedings of the IEEE International Conference on Computer Vision.

[22] Liu W., Anguelov D., Erhan D., Szegedy C., Reed S., Fu C.-Y., Berg A.C. Ssd: Single shot multibox detector. Proceedings of the European Conference on Computer Vision.

[23] Bewley A., Ge Z., Ott L., Ramos F., Upcroft B. Simple online and real-time tracking. Proceedings of the 2016 IEEE International Conference on Image Processing (ICIP).

[24] Wojke N., Bewley A., Paulus D. Simple online and real-time tracking with a deep association metric. Proceedings of the 2017 IEEE International Conference on Image Processing (ICIP).

[25] Feichtenhofer C., Pinz A., Zisserman A. Detect to Track and Track to Detect. Proceedings of the IEEE International Conference on Computer Vision.

[26] Zhu J., Yang H., Liu N., Kim M., Zhang W., Yang M.-H. Online multi-object tracking with dual matching attention networks. Proceedings of the European Conference on Computer Vision (ECCV).

[27] Son J., Baek M., Cho M., Han B. Multi-object tracking with quadruplet convolutional neural networks. Proceedings of the IEEE Conference on Computer Vision and Pattern Recognition.

[28] Qin X., Zhang Z., Huang C., Dehghan M., Zaiane O.R., Jagersand M. (2020). U^2^-Net: Going deeper with nested U-structure for salient object detection. Pattern Recognit..

[29] Zeng Y., Lin Z., Lu H., Patel V.M. Cr-fill: Generative image inpainting with auxiliary contextual reconstruction. Proceedings of the IEEE/CVF International Conference on Computer Vision.

[30] Kang B., Liu Z., Wang X., Yu F., Feng J., Darrell T. Few-shot object detection via feature reweighting. Proceedings of the IEEE/CVF International Conference on Computer Vision.

[31] Mangla P., Kumari N., Sinha A., Singh M., Krishnamurthy B., Balasubramanian V.N. Charting the right manifold: Manifold mixup for few-shot learning. Proceedings of the IEEE/CVF Winter Conference on Applications of Computer Vision.

[32] Bernardin K., Stiefelhagen R. (2008). Evaluating multiple object tracking performance: The clear mot metrics. EURASIP J. Image Video Processing.

[33] Zagoruyko S., Komodakis N. (2016). Wide residual networks. arXiv.

[34] Milan A., Leal-Taixé L., Reid I., Roth S., Schindler K. (2016). MOT16: A benchmark for multi-object tracking. arXiv.

[35] Zhang Y., Sun P., Jiang Y., Yu D., Yuan Z., Luo P., Liu W., Wang X. (2021). ByteTrack: Multi-Object Tracking by Associating Every Detection Box. arXiv.

[36] Bochinski E., Eiselein V., Sikora T. High-speed tracking-by-detection without using image information. Proceedings of the 2017 14th IEEE International Conference on Advanced Video and Signal Based Surveillance (AVSS).

[37] Bochinski E., Senst T., Sikora T. Extending IOU based multi-object tracking by visual information. Proceedings of the 2018 15th IEEE International Conference on Advanced Video and Signal Based Surveillance (AVSS).

[38] Tuia D., Kellenberger B., Beery S., Costelloe B.R., Zuffi S., Risse B., Mathis A., Mathis M.W., van Langevelde F., Burghardt T. (2022). Perspectives in machine learning for wildlife conservation. Nat. Commun..

[39] Feng J., Li J. (2022). An Adaptive Embedding Network with Spatial Constraints for the Use of Few-Shot Learning in Endangered-Animal Detection. ISPRS Int. J. Geo-Inf..

[40] Hill S.P., Broom D.M. (2009). Measuring zoo animal welfare: Theory and practice. Zoo Biol..

[41] Watters J., Krebs B., Pacheco E., Kaufman A., Bashaw M., Maples T. (2019). Measuring welfare through behavioral observation and adjusting it with dynamic environments. Scientific Foundations of Zoos and Aquariums: Their Roles in Conservation and Research.

[42] Skovlund C.R., Kirchner M.K., Moos L.W., Alsted N., Manteca X., Tallo-Parra O., Stelvig M., Forkman B. (2021). A critical review of animal-based welfare indicators for polar bears (Ursus maritimus) in zoos: Identification and evidence of validity. Anim. Welf.

